# Effects of Straw Incorporation on Soil Organic Matter and Soil Water-Stable Aggregates Content in Semiarid Regions of Northwest China

**DOI:** 10.1371/journal.pone.0092839

**Published:** 2014-03-24

**Authors:** Peng Zhang, Ting Wei, Zhikuan Jia, Qingfang Han, Xiaolong Ren, Yongping Li

**Affiliations:** 1 The Chinese Institute of Water-Saving Agriculture, Northwest A&F University, Yangling, Shaanxi, China; 2 Key Laboratory of Crop Physi-Ecology and Tillage Science in Northwestern Loess Plateau, Ministry of Agriculture, Northwest A&F University, Yangling, Shaanxi, China; 3 Guyuan Institute of Agricultural Sciences, Guyuan, Ningxia, China; Institute for Plant Protection (IPP), CNR, Italy

## Abstract

The soil degradation caused by conventional tillage in rain-fed areas of northwest China is known to reduce the water–use efficiency and crop yield because of reduced soil porosity and the decreased availability of soil water and nutrients. Thus, we investigated the effects of straw incorporation on soil aggregates with different straw incorporation rates in semiarid areas of southern Ningxia for a three-year period (2008–2010). Four treatments were tested: (i) no straw incorporation (CK); (ii) incorporation of maize straw at a low rate of 4 500 kg ha^−1^ (L); (iii) incorporation of maize straw at a medium rate of 9000 kg ha^−1^ (M); (iv) incorporation of maize straw at a high rate of 13 500 kg ha^−1^ (H). The results in the final year of treatments (2010) showed that the mean soil organic carbon storage of the 0–60 cm soil layers were significantly (*P<*0.05) increased with H, M, and L, by 21.40%, 20.38% and 8.21% compared with CK, respectively. Straw incorporation increased >0.25 mm water-stable macroaggregates level, geometric mean diameter, mean weight diameter and the aggregate stability, which were ranked in order of increasing straw incorporation rates: H/M > L > CK. Straw incorporation significantly (*P<*0.05) reduced the fractal dimension in the 0–40 cm soil layers compared with CK. Our results suggest that straw incorporation is an effective practice for improving the soil aggregate structure and stability.

## Introduction

Soil infertility [1], soil erosion, and water deficiency [2] are the major factors that limited crop growth in semiarid areas of northwest China. The rates of crop straw use for fuel and forage have declined significantly since the 1980s and crop straw is increasingly burned after the harvest, which leads to high losses of soil organic substances [3], [4], and increased emission of CO_2_ that pollute the environment [5]. Furthermore, this practice has led to the degradation of the agricultural ecological environment [6].

The soil organic matter (SOM) content is one of the major factors that affects soil properties and functions including a range of physical characteristics such as the water-holding capacity [7], water infiltration [8], and aggregate stability [9]. SOM is considered to be a major binding agent that stabilizes soil aggregates [10], [11]. Soil aggregates are the basic units of the soil structure [12], which are composed of primary particles and binding agents that determine the microbial biomass and mineral nutrient reserves [13]–[15]. These soil properties are also affected by soil organic matter decomposition processes [16], [17].

Many studies have shown that crop straw is rich in organic material and soil nutrients, so it is increasingly considered to be an important natural organic fertilizer [18]–[20]. Straw can be incorporated to soil either directly or indirectly, which can promote the production of a favorable soil environment. Straw also maintains the physicochemical condition of the soil and improves the overall ecological balance of the crop production system [20], [21]. Nelson [22] and Wilhelm *et al.* [23] showed that the incorporation of crop residues into soil significantly prevented soil erosion and enhanced the soil quality. Sonnleitner *et al.* [24] found that straw incorporation also improved the aggregate stability and other soil properties compared with farmyard manure. Mulumba and Lal [25] also reported that the addition of crop residues to cultivated soil had positive effects on the soil porosity, available water content, soil aggregation, and bulk density. Bhagat and Verma [26] showed that the incorporation of crop straws for five years significantly increased the crop yield and improved the soil properties.

The soil improvement effect of straw incorporation has been recognized widely [23]–[25] but information is still limited on the responses of the SOC and water-stable aggregates under different rates of straw incorporation, particularly in the loessal soil in semiarid areas of northwest China. The theory and technique of straw incorporation in this region have also not been reported. Thus, the present study investigated the effects of different crop straw application rates combined with conventional planting on SOC, the >0.25 mm water-stable macroaggregate rate, and various soil properties in the southern Ningxia region of China.

## Materials and Methods

### Ethics Statements

The study was carried out on the private land, we rent the farmland from the local farmers, and contracts and deeds are signed. No specific permissions were required in this area to run the experiment as the study sites are farming area without any protection zone, and the farming activities won't hurt the local animals. And we only plant the grain crop in the field, so the field studies did not involve endangered or protected species.

### Site description

The experiment was conducted between 2008 and 2010 at the Dryland Agricultural Research Station, Pengyang County, Ningxia, China (106°45'N, 35°79'E and 1800 m a.s.l.). The experimental area was in a hilly and gully region of the Loess Plateau, which was characterized by a semiarid, warm temperate, continental monsoon climate. The average annual rainfall was 435 mm, which fell mainly from June to September. The annual mean evaporation was 1050 mm and the annual temperature average was 8.1°C with a frost-free period of 155 days.

**Figure 1:**
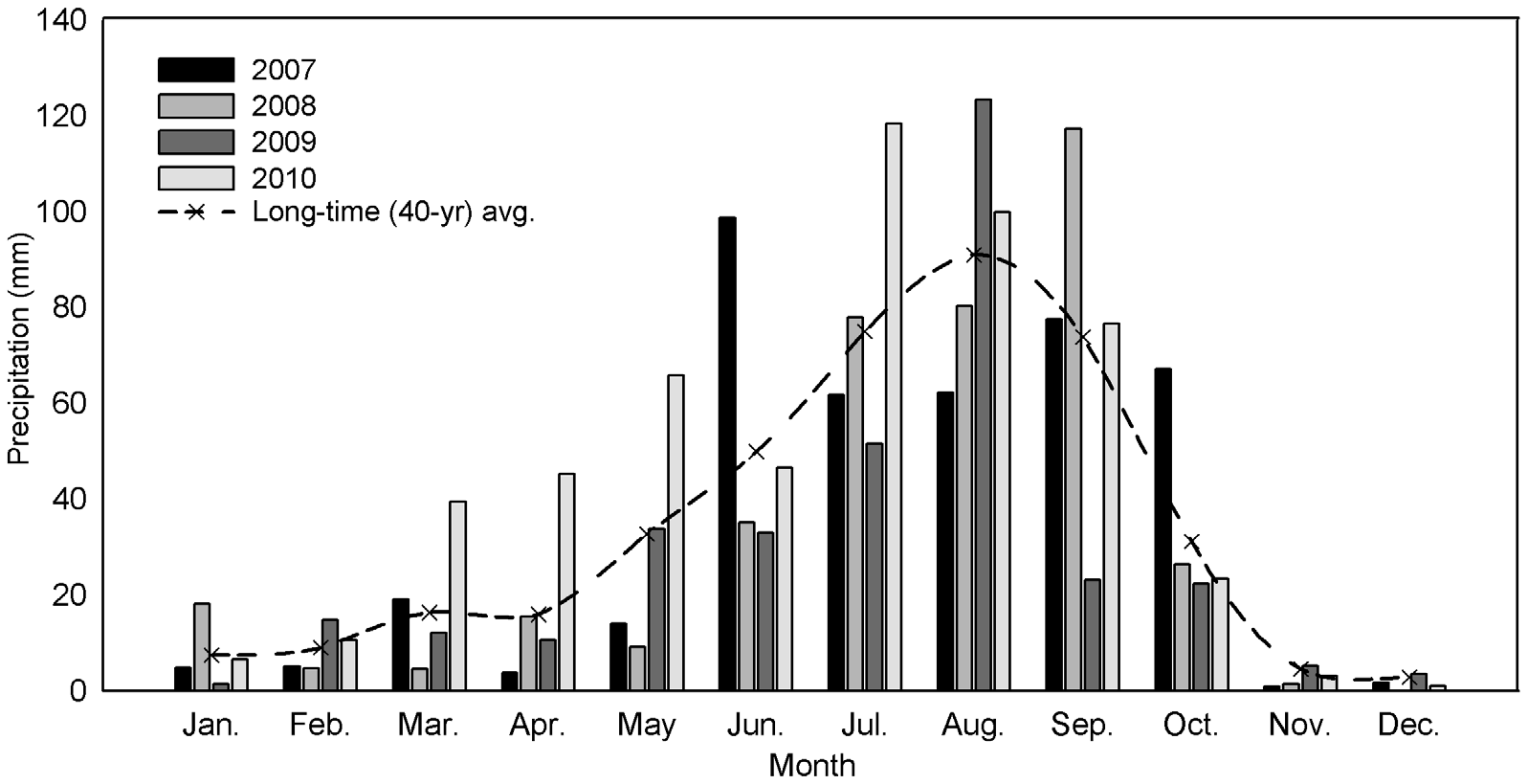
Distribution of mean monthly precipitation at the experimental site during 2007–2010.

Rainfall during the experimental period was measured using an automatic weather station (WS-STD1, England) at the experimental site. Monthly precipitation distributions during the experimental period are shown in Fig. 1. The total precipitation for 2008, 2009, and 2010 was 390.9, 335.2, and 537 mm, while the precipitation during the maize-growing season was 362, 298.2, and 476.1 mm, respectively.

The soil at the experimental site was a loessal soil with a pH of 8.5. In the 0–40 cm soil layer, the organic matter, total N, P, and K were 8.32 g kg^−1^, 0.61 g kg^−1^, 0.58 g kg^−1^, and 5.4 g kg^−1^, respectively, while the available N, P, and K were 46.25 mg kg^−1^, 10.41 mg kg^−1^, and 104.82 mg kg^−1^. In 2007, the site was planted with maize prior to the experiment

The experimental field was flat and, according to the FAO/UNESCO Soil Classification [27], the soil was a Calcic Cambisol (sand 14%, silt 26%, and clay 60%) with low fertility. The key physical properties of the soil layers (0–40 cm depth) are shown in Table 1.

**Table 1:** Physical properties of the tilth soil (0–40 cm depth) in the experimental site.

Depth (cm)	Bulk density (g cm^−3^)	Aggregate size (%)					
		>5 mm	5–2 mm	2–1 mm	1–0.5 mm	0.5–0.25 mm	<0.25 mm
0–10	1.33	0.1	0.27	2.25	4.5	4.47	88.41
10–20	1.33	0.15	0.19	1.38	4.2	4.15	89.93
20–30	1.36	0.1	0.28	1.41	4.02	3.36	90.83
30–40	1.38	0.1	0.22	1.07	3.29	3.8	91.52

### Experimental design and field management

The experiment used a randomized block design with three replicates. Each plot was 3 m wide and 6 m long. The experiment included four straw incorporation rate treatments: (i) no straw incorporation (CK); (ii) incorporation of maize straw at a low rate of 4 500 kg ha^−1^ (L); (iii) incorporation of maize straw at a medium rate of 9000 kg ha^−1^ (M); (iv) incorporation of maize straw at a high rate of 13 500 kg ha^−1^ (H).

The maize straws were mixed manually with the top 25 cm of soil in the field. Before mixing with the soil, the maize straws were chopped into 5 cm pieces and then applied to the soil six months before the crop was planted to facilitate decomposition of the straw. The straw was incorporated into the soil layer on 15 October 2007 and after the crop harvests during 2008–2010.

Ten days before sowing, a basis fertilizer containing 102 kg N ha^−1^ and 90 kg P ha^−1^, was spread evenly over the each plot and plowed into soil layer. Maize (cv. Shendan 16) was sown at a rate of 5.25 seed m^−2^ on 18 April 2008, 15 April 2009 and 20 April 2010 using a holesowing (3 cm in diameter) machine. An additional 102 kg N ha^−1^ was applied as a top dressing in late June. And on 7 October 2008, 5 October 2008, and 10 October 2010. No irrigation was provided during the experimental years. Manual weeding was performed throughout the experiment.

### Sampling and measurement

Rainfall data were recorded using a standard weather station located at the experimental site. After the maize harvest in 2008 and 2010, soil samples were collected for the four incorporation treatments. A soil sample was collected from each plot at depths of 0–20 cm, 20–40 cm and 40–60 cm to determine the soil organic matter. A similar soil sample was collected at depths of 0–10 cm, 10–20 cm, 20–30 cm and 30–40 cm to determine the aggregate stability. The soil samples were collected from four points in each plot replicate and mixed to produce a composite sample. Each soil samples was passed through an 8 mm sieve by gently breaking the soil clods, whereas pebbles and stable clods >8 mm were discarded. Soil samples were air-dried for 24 h in the laboratory before analysis.

Soil organic carbon was determined by the K_2_Cr_2_O_7_–H_2_SO_4_ digestion method, and SOM content was calculated as a portion of SOC which has been described by Wang *et al.* [28].
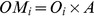
(1)

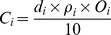
(2)


where 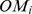 is the soil organic matter content (g kg^−1^), 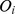
is the soil organic carbon content (g kg^−1^), 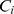 is the soil organic carbon storage (Mg C ha^−1^), 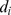
is the soil depth (cm), 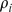 is the soil bulk density (g cm^−3^), 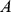 is Van Bemmelen coefficient (A = 2) [29].

The size distribution of water-stable aggregates was determined by placing a soil sample on a stack of sieves (5,2, 1, 0.5 and 0.25 mm) fitted with a soil aggregate analyzer (Japan, QD24–DIK–2001). The stacked sieves were immersed in water and moved up and down by 3.5 cm at a frequency of 30 cycles 60s^−1^ for 15 min. The proportions of aggregates that measured >5, 5–2, 2–0.5, 0.5–0.25 and <0.25 mm were calculated [30].

And the proportions of aggregates were used to calculate the water-stable macroaggregates content with a diameter of >0.25 mm [31], mean weight diameter (MWD) [32], [33], geometric mean diameter (GMD) [34], and the soil aggregate stability (WSAR) [35]. These parameters were calculated as follows:
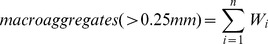
(3)

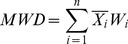
(4)

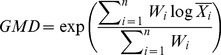
(5)

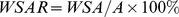
(6)


where 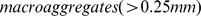 is the volume of soil particles with a diameter of >0.25 mm, 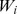
is weight of the aggregates in that size range as a fraction of total dry weight of the sample analysed, and 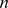 is number of sieves, 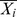 is the mean diameter of aggregates over each sieve size, 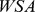 is the mass of 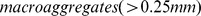, and 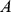 is the mass of the soil aggregates with a diameter of >0.25 mm.

Fractal dimension D were then obtained to describe the characteristics of soil aggregate size distribution. As suggested by Tyler and Wheatcraft [36] and Zhang *et al*., [37], the volume of soil with particle diameter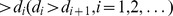 is defined as:
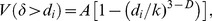
(7)


where 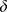 is yard measure, 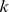 and 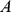 are the constants representing size and shape, respectively, and 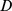 is the fractal dimension. For a given soil, 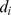 represents the average value of soil particles between 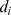 and 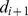.Generally, variations of particle density 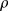 among different soil particles could be ignored. And hence 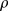 is a constant. Therefore, another expression (Eq. (8)) is derived from Eq. (7):
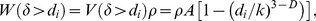
(8)


where 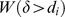 is the cumulative mass of particles with sizes 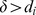, and 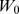 is the total mass of any sizes of soil particles. The fractal equation, reflecting the relationship between the mass distributions of soil particles and average particle diameter, can be obtained as follows:
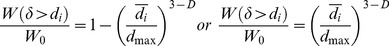
(9)


Then after regression analysis between 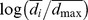 and 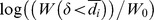, the fractal dimension 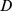 can be calculated.

### Statistical analysis

Statistical analysis was carried out by using the SPSS 13.0 (Statistical Package for the Social Sciences) package. The effects of treatments on the measured parameters were evaluated using a one-way ANOVA. Duncan’s new multiple range test was used to calculate the least significant difference (LSD) between means when F-values were significant. In all cases, differences were deemed to be significant if *P*<0.05.

## Results

### Soil organic carbon storage (SOC)

**Figure 2:**
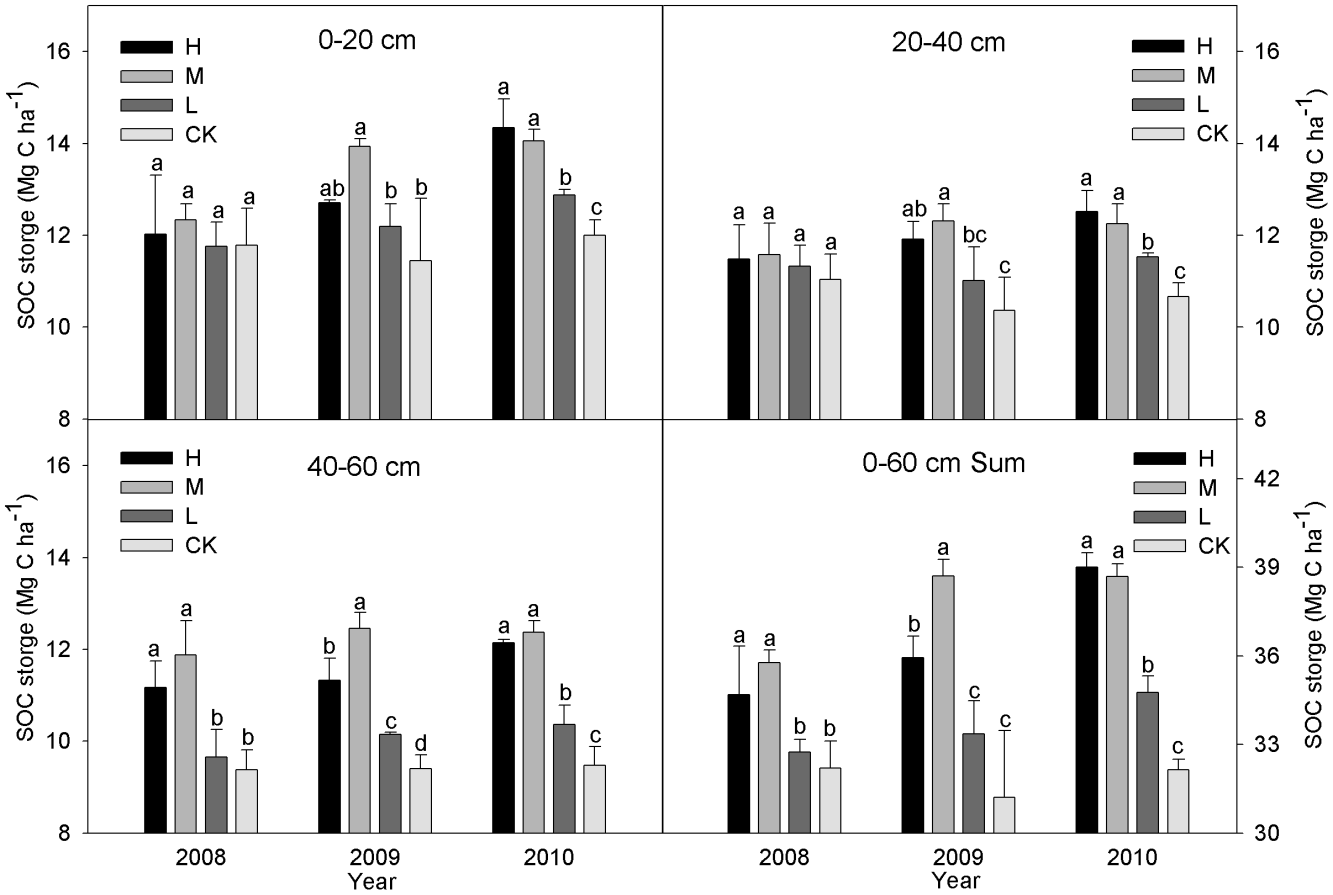
Soil organic carbon storage with different straw incorporation treatments (Mg C ha^−1^). Note: CK, no straw incorporation; L, incorporation of straw at a low rate of 4 500 kg ha^−1^ maize straw; M, incorporation of straw at a medium rate of 9 000 kg ha^−1^ maize straw; H, incorporation of straw at a high rate of 13 500 kg ha^−1^ maize straw; Sum, the sum value of the 0–60 cm soil layers; Bars with different lower case letters indicate significant differences at *P*<0.05.

The effect of straw incorporation on SOC storage is shown in Fig. 2, where the soil organic carbon storage increased with the straw incorporation. The sum of SOC storage in 0–60 cm layers with the three incorporation treatments were higher than CK, i.e., 7.71% (*P<*0.05), 11.14% (*P<*0.05) and 1.70% in 2008, 15.15% (*P<0.05*), 24.00% (*P<0.05*) and 6.86% in 2009, and 21.40% (*P<0.05*), 20.38% (*P<0.05*) and 8.21% (*P<*0.05) in 2010, respectively. The SOC storage increased with the number of years of incorporation, i.e., the SOC storage (0–60 cm depth) in 2010 had increased by 6.19–12.48% compared with 2008, and decreased with the soil layer depth, i.e., by 3.75–25.68% in 2008, 11.85–21.70% in 2009 and 13.51–26.64% in 2010. The SOC storage of H and M was slightly higher than CK in 2008, although the difference was significant in 40–60 cm layer only. In 2009, compared with CK, H and M significantly increased the SOC storage by 11.01% and 21.74% (*P<*0.05) in 0–20 cm layer, 14.94% and 18.81% (*P<*0.05) in 20–40 cm layer, and 20.40% and 32.47% (*P<*0.05) in 40–60 cm layer, respectively, and L was significantly increased by 7.89% (*P<*0.05) in 40–60 cm layer only. There was a significant difference between straw incorporation treatments and CK for each of the three soil layers in 2010, but there were no significant differences between H and M.

### Water-stable macroaggregates (>0.25 mm)

The effects of straw incorporation on >0.25 mm macroaggregates are shown in Table 2. The mean >0.25 mm macroaggregates contents of the 0–40 cm layers of incorporation treatments were significantly (P<0.05) higher than CK during 2008–2010, i.e., 8.91–41.14%, 15.13–49.94%, and 17.64–66.58%, respectively. The >0.25 mm macroaggregates content also decreased with the soil layer depth, and increased with the number of years of incorporation,, i.e., the H, M, L and CK in 2010 was 24.72% (*P*<0.05), 39.28% (*P*<0.05), 16.49% and 7.81% higher than that in 2008. Compared with CK, the >0.25 mm macroaggregates content of three straw incorporation treatments in the 0–10 cm layer was increased by 10.75–46.60% in 2008, 6.56–53.13% in 2009, and 7.53–63.44% in 2010, respectively, while the differences between L and CK were not significant (*P>*0.05) during 2008–2010. The >0.25 mm macroaggregates content showed the same trends in the 10–20 and 20–30 cm layers, and the H and M treatments had the significant difference (*P*<0.05) with CK. The H, M and L increased by 29.05% (*P*<0.05), 16.55% and 6.87% in 2008, 38.94% (*P*<0.05), 24.96% and 14.69% in 2009, and 50.09% (*P*<0.05), 54.29% (*P*<0.05), 21.89% (*P*<0.05) in 2010, respectively. A linear correlation was found between the >0.25 mm macroaggregates and soil organic carbon (*R* ^2^>0.64, *P*<0.01).

**Table 2:** WR_0.25_ with different straw incorporation treatments (%).

Year	Treatment	Soil depth (cm)				0–40 AVG
		0—10	10—20	20—30	30—40	
2008	H	13.37a_1_±0.33^B2^	10.57a±1.28_3_^A^	7.72ab±0.27^B^	7.33a±0.51^A^	9.75a±0.31^B^
	M	11.79b±1.05^A^	9.28a±0.60^B^	7.97a±0.65^A^	6.62ab±0.85^B^	8.91b±0.26^B^
	L	10.10c±0.46^A^	7.39b±0.58^A^	6.53bc±1.08^A^	6.07ab±0.81^A^	7.52c±0.16^A^
	CK	9.12c±0.70^A^	6.93b±0.78^A^	5.89c±0.57^A^	5.68b±0.63^A^	6.91d±0.44^A^
2009	H	14.64a±0.49^AB^	11.46a±0.17^A^	9.51a±0.26^AB^	7.85a±1.00^A^	10.86a±0.19^AB^
	M	14.93a±0.89^A^	11.22a±0.14^AB^	9.34a±0.32^A^	7.06ab±1.46^AB^	10.64a±0.27^AB^
	L	10.39b±0.26^A^	9.48b±1.49^A^	7.02b±1.18^A^	6.48ab±1.10^A^	8.34b±0.32^A^
	CK	9.75b±0.26^A^	7.18c±0.73^A^	6.40b±1.00^A^	5.65b±0.35^A^	7.25c±0.10^A^
2010	H	15.81a±1.20^A^	12.76a±0.44^A^	11.50a±0.20^A^	8.57a±0.05^A^	12.16a±0.59^A^
	M	16.72a±1.36^A^	12.81a±0.05^A^	11.30a±1.10^A^	8.81a±0.44^A^	12.41a±0.72^A^
	L	11.00b±0.78^A^	9.06b±0.81^A^	8.03b±2.14^A^	6.96b±0.81^A^	8.76b±0.66^A^
	CK	10.23b±0.46^A^	7.79b±0.23^A^	6.07b±0.46^A^	5.71c±0.23^A^	7.45c±0.25^A^

Note: CK, no straw incorporation; L, incorporation of straw at a low rate of 4 500 kg ha^−1^ maize straw; M, incorporation of straw at a medium rate of 9 000 kg ha^−1^ maize straw; H, incorporation of straw at a high rate of 13 500 kg ha^−1^ maize straw; AVG, the mean value of the 0–40 cm soil layers.

1 Values followed by the same lowercase letter in the same line are not significantly different according to Duncan’s multiple range test (*P*<0.05) between the four straw incorporation treatments in the same year.

2 Values followed by the same uppercase letter in the same line are not significantly different according to Duncan’s multiple range test (*P*<0.05) between the different years of the same straw incorporation treatment.

3 Means ± standard deviations.

### Mean weight diameter (*MWD*) and Geometric mean diameter (*GMD*)

**Figure 3:**
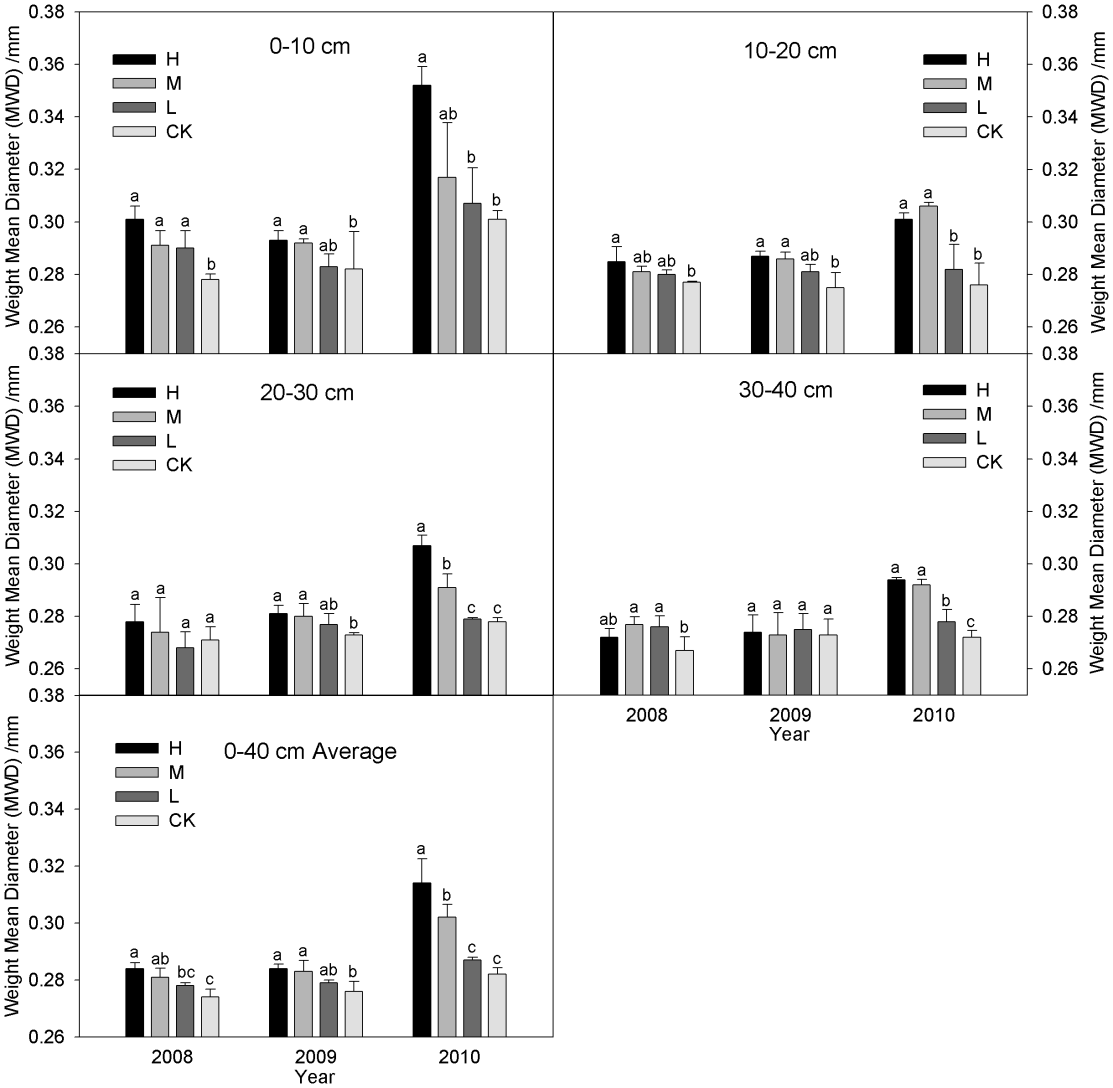
MWD values with wet sieving under the different straw incorporation treatments. Note: MWD, weight mean diameter; CK, no straw incorporation; L, incorporation of straw at a low rate of 4 500 kg ha^−1^ maize straw; M, incorporation of straw at a medium rate of 9 000 kg ha^−1^ maize straw; H, incorporation of straw at a high rate of 13 500 kg ha^−1^ maize straw; Average, the mean value of the 0–40 cm soil layers. Bars with different lower case letters indicate significant differences at *P*<0.05.

**Figure 4:**
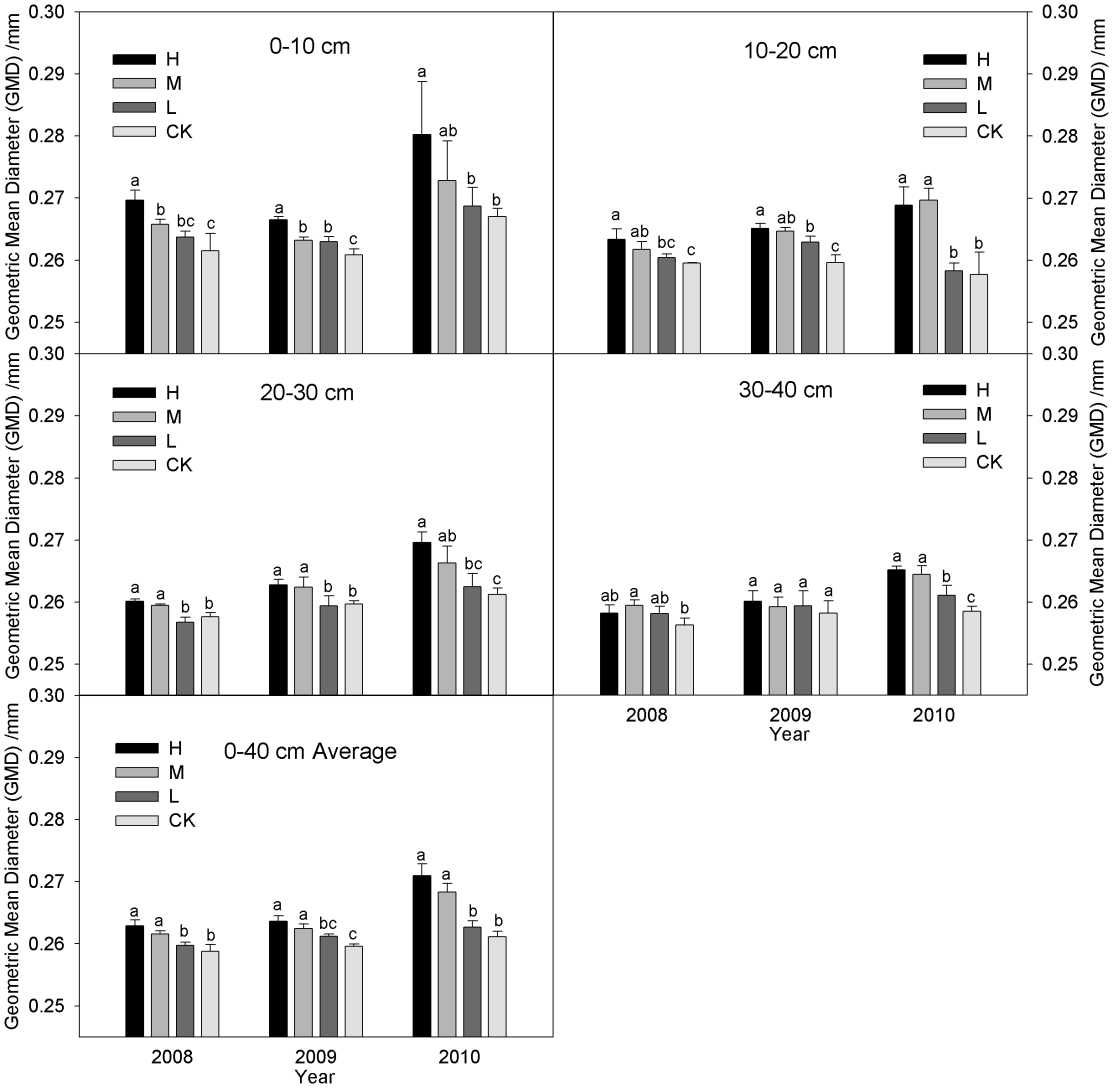
GMD values with wet sieving under the different straw incorporation treatments. Note: GMD, geometric mean diameter; CK, no straw incorporation; L, incorporation of straw at a low rate of 4 500 kg ha^−1^ maize straw; M, incorporation of straw at a medium rate of 9 000 kg ha^−1^ maize straw; H, incorporation of straw at a high rate of 13 500 kg ha^−1^ maize straw; Average, the mean value of the 0–40 cm soil layers. Bars with different lower case letters indicate significant differences at *P*<0.05.

Fig. 3 & 4 show that the *MWD* and *GMD* values with the three incorporation treatments increased significantly throughout the three-year study. The average *MWD* and *GMD* values under the incorporation treatments were higher in the 0–40 cm layers than CK during 2008–2010, i.e., 1.46–3.65% and 0.39–1.54% in 2008, 1.09–2.90% and 0.62–1.55% in 2009, 1.77–11.35% and 0.77–3.83% in 2010, respectively. There was no significant difference (*P*>0.05) between L and CK during the study period. The *MWD* and *GMD* values decreased with the soil layer depth and increased with the number of years of incorporation, i.e., the H, M, L and CK in 2010 was 10.56% (*P*<0.05) and 3.04% (*P*<0.05), 7.47% (*P*<0.05) and 2.29% (*P*<0.05), 3.24% and 1.15%, and 2.92% and 0.77% higher than that in 2008. The *MWD* and *GMD* values of treatments in the 0–10 and 10–20 cm layers were ranked in the order: H > M > L > CK. The H and M levels were significantly higher than CK while H and M levels were similar during 2008–2010 (Fig. 3–4). The *MWD* and *GMD* exhibited the same trends in 20–30 cm layers and there were no significant (*P*>0.05) differences among incorporation treatments during 2008–2009, the *MWD* values with the H were significantly (P<0.05) higher than the other three treatments during 2010, i.e., by 5.50%, 10.04%, and 10.43%, respectively. The *GMD* did not differ significantly among the four treatments, with the exceptions of H and L, and H and CK in 2010. The *MWD* and *GMD* values in the 30–40 cm layers under the incorporation treatments were increased by 1.87%, 3.75% (*P*<0.05), 3.37% (*P*<0.05) and 0.76%, 1.76% (*P*<0.05), 0.78% in 2008, 0.37%, 0.01%, 0.73% and 0.73%, 0.45%, 0.45% in 2009, 8.09% (*P*<0.05), 7.35% (*P*<0.05), 2.21% (*P*<0.05) and 2.32% (*P*<0.05), 1.93% (*P*<0.05), 0.77% (*P*<0.05) in 2010, compared with CK, respectively. There was no significant difference (*P*>0.05) between H and M during the study period.

### Soil aggregate stability *(WSAR)*

The straw incorporation significantly affected the *WSAR* after three years (Table 3). The *WSAR* values in the 0–40 cm layers of incorporation treatments was significantly higher than CK during 2008–2010, i.e., 0.34–19.80%, 9.65–33.79%, and 6.16–35.08%, respectively. The *WSAR* also decreased with the soil layer depth, while it increased with the number of years of incorporation. The *WSAR* in the 0–10 cm layers increased significantly with the amount of straw and the *WSAR* was highest with H throughout 2008–2010, i.e., the *WSAR* significantly (*P*<0.05) increased by 28.35%, 28.93% and 25.78% compared with L, respectively, and by 33.62%, 31.35% and 32.89% (*P*<0.05) compared with CK, respectively. Compared with L and CK, the WSAR values with M increased significantly by 20.08–24.67% and 26.87–29.78% during 2008–2010, respectively, but there were no significant (*P*>0.05) differences between H and M, and L and CK. With the four treatments, the *WSAR* values in the 10–20 and 20–30 cm layers were lower than the 0–10 cm layers, which were ranked in the order: H > M > L > CK. There were no significant differences (*P*>0.05) among the four treatments in the 30–40 cm layers throughout the three years, and the *WSAR* maintained at 7.50–10.45%.

**Table 3:** Effects on the soil aggregate stability rate with different straw incorporation treatments (%).

Year	Treatments	Soil Depth (cm)				0–40 AVG
		0–10	10–20	20–30	30–40	
2008	H	21.86a_1_±0.89^A2^	16.29a±0.41_3_^A^	10.83a±0.54^B^	9.19a±0.65^A^	14.60a±0.33^A^
	M	19.62ab±1.09^A^	15.31b±0.62^A^	11.16a±0.82^A^	8.47b±0.21^A^	13.70b±0.62^A^
	L	17.83bc±1.56^A^	12.88c±0.24^A^	9.70b±0.64^A^	8.00bc±0.42^A^	11.83c±0.18^A^
	CK	16.45c±2.42^A^	12.52c±0.58^A^	9.39b±0.80^A^	7.53c±0.37^A^	11.79c±0.45^A^
2009	H	20.32a±0.27^A^	16.14a±0.54^A^	13.15a±0.34^AB^	9.74a±0.99^A^	14.84a±0.23^A^
	M	20.02a±0.46^A^	15.41a±0.50^A^	12.61a±0.41^A^	8.66ab±1.28^A^	14.18b±0.22^A^
	L	15.76b±0.49^A^	14.45a±1.78^A^	10.16b±1.36^A^	8.28ab±1.01^A^	12.16c±0.35^A^
	CK	15.47b±0.30^A^	11.81b±0.83^A^	9.59b±1.10^A^	7.50b±0.41^A^	11.09d±0.05^A^
2010	H	20.19a±1.93^A^	16.48a±1.73^A^	15.24a±0.12^A^	10.45a±0.11^A^	15.59a±0.26^A^
	M	20.87a±0.68^A^	15.78a±1.32^A^	13.99a±1.23^A^	10.08a±0.43^A^	15.18a±0.64^A^
	L	15.72b±0.72^A^	13.39a±1.81^A^	12.53b±0.46^A^	8.66b±0.77^A^	12.58b±1.00^A^
	CK	15.11b±0.12^A^	12.79a±1.70^A^	12.00b±0.76^A^	7.52c±0.26^A^	11.85b±1.32^A^

Note: CK, no straw incorporation; L, incorporation of straw at a low rate of 4 500 kg ha^−1^ maize straw; M, incorporation of straw at a medium rate of 9 000 kg ha^−1^ maize straw; H, incorporation of straw at a high rate of 13 500 kg ha^−1^ maize straw; AVG, the mean value of the 0–40 cm soil layers.

1 Values followed by the same lowercase letter in the same line are not significantly different according to Duncan’s multiple range test (*P*<0.05) between the four straw incorporation treatments in the same year.

2 Values followed by the same uppercase letter in the same line are not significantly different according to Duncan’s multiple range test (*P*<0.05) between the different years of the same straw incorporation treatment.

3 Means ± standard deviations.

### Fractal dimension (D)

**Figure 5:**
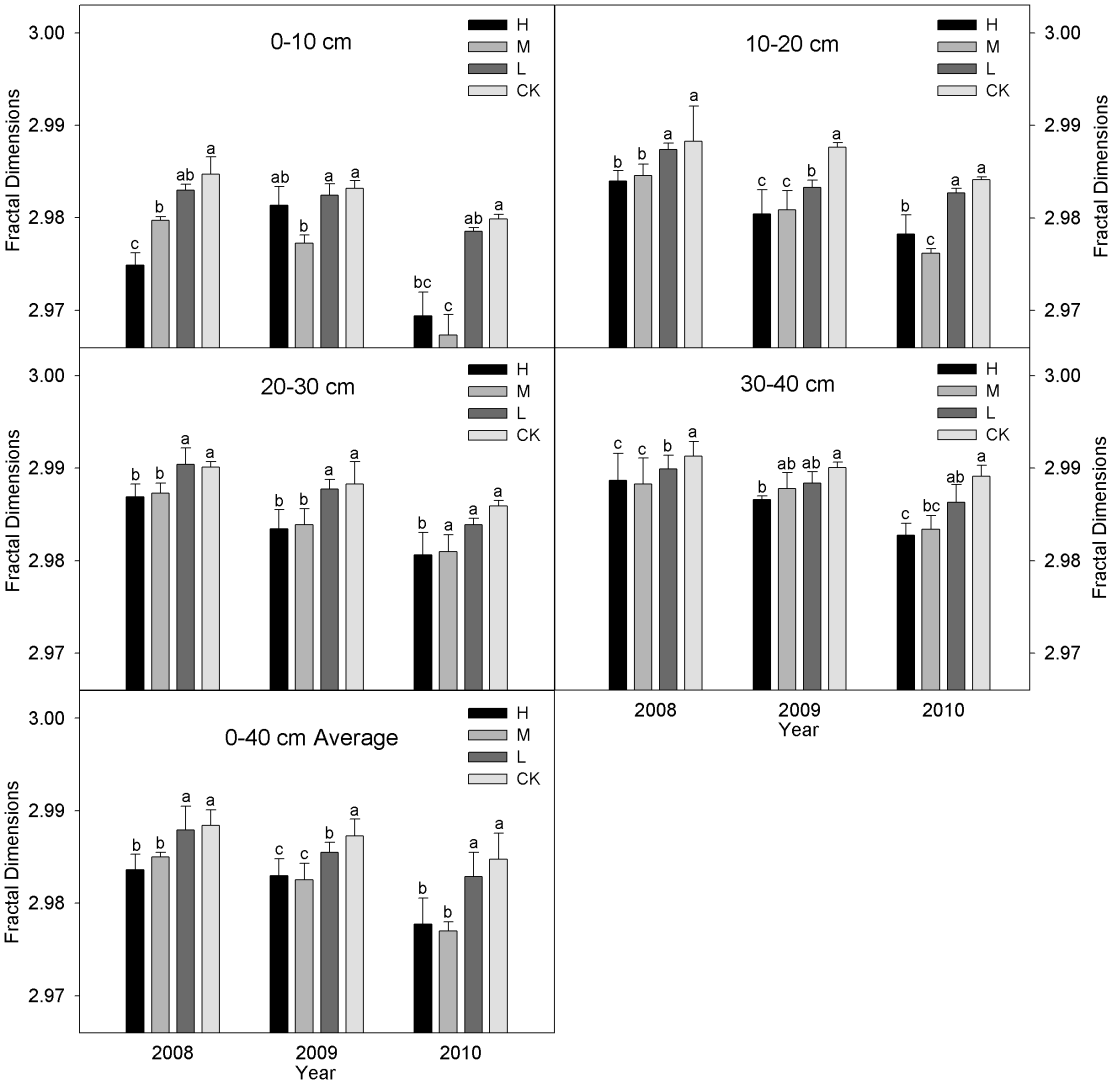
Fractal dimensions (D) of soil aggregates with different straw incorporation treatments. Note: CK, no straw incorporation; L, incorporation of straw at a low rate of 4 500 kg ha^−1^ maize straw; M, incorporation of straw at a medium rate of 9 000 kg ha^−1^ maize straw; H, incorporation of straw at a high rate of 13 500 kg ha^−1^ maize straw; Average, the mean value of the 0–60 cm soil layers; Bars with different lower case letters indicate significant differences at *P*<0.05; 0.92<R^2^<0.99.

Fig. 5 shows that the fractal dimensions with the three incorporation treatments decreased significantly (*P*<0.05) after three years of straw incorporation (the R^2^ of fitting curve is: 0.92∼0.99). The fractal dimensions (0–40 cm layer) with the incorporation treatments were lower than those of CK during 2008–2010, i.e., 0.16% (*P*<0.05), 0.11% (*P*<0.05) and 0.02% in 2008, 0.14% (*P*<0.05), 0.16% (*P*<0.05) and 0.06% (*P*<0.05) in 2009, and 0.24% (*P*<0.05), 0.26% (*P*<0.05) and 0.06% in 2010, respectively. The fractal dimensions also increased with the soil layer depth and decreased with the number years of incorporation, i.e., the H, M, L and CK in 2010 was 0.20% (*P*<0.05), 0.27% (*P*<0.05), 0.17% (*P*<0.05) and 0.12% higher than that in 2008. The fractal dimensions in the 0–10 cm layers with the four treatments were ranked in the order: H<M<L<CK in 2008. The fractal dimensions with H and M were significantly (*P*<0.05) lower than CK, i.e., 0.33%, 0.17% and 0.06%, respectively. However, the ranking was M<H<L<CK during 2009–2010, i.e., 0.06%, 0.20% (*P*<0.05), 0.02% in 2009, 0.35% (*P*<0.05), 0.42% (*P*<0.05) and 0.04% in 2010, respectively. While the differences between L and CK were not significant (*P*>0.05) during 2008–2010. Compared with L and CK, the fractal dimensions in the 10–20 cm layers under H was significantly (*P*<0.05) reduced the fractal dimensions by 0.11% and 0.14% in 2008, and 0.10% and 0.24% in 2009,, while M significantly (*P*<0.05) reduced by 0.09% and 0.12% in 2008, and 0.08% and 0.23% in 2009, respectively. The fractal dimension was lowest with M during 2010, i.e., it was decreased by 0.22% (*P*<0.05) compared with L, and by 0.27% (*P*<0.05) compared with CK. The differences among the four treatments decreased gradually in the 20–40 cm layer and the fractal dimension remained at 2.987∼2.991.

**Figure 6:**
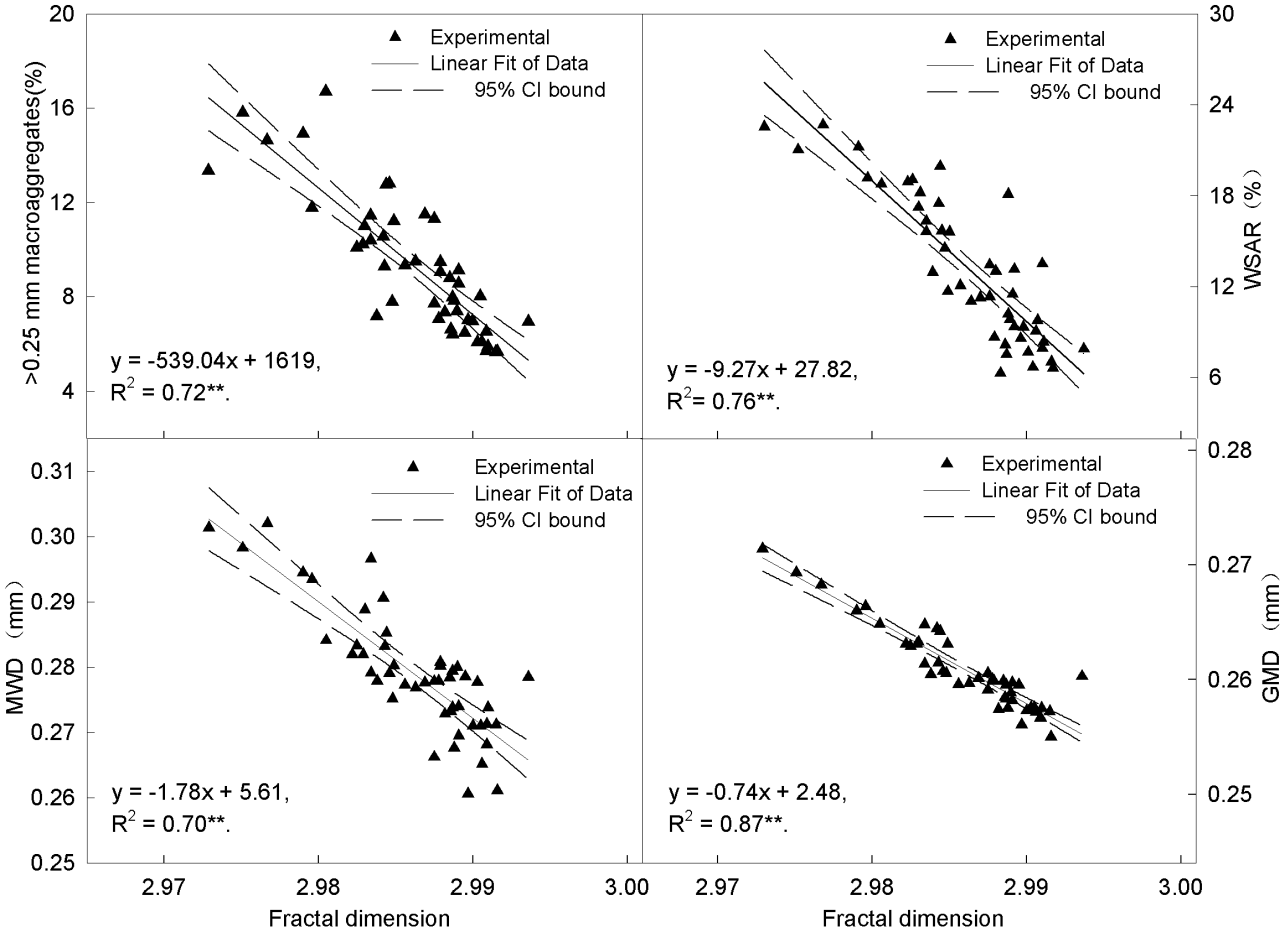
Correlations between D and >0.25 mm macroaggregates, MWD, GMD, WSAR of the soil aggregates. Note: MWD, weight mean diameter of soil aggregates; GMD, geometric mean diameter of soil aggregates; WSAR, soil aggregate stablility rate; D, Fractal dimension of soil aggregates. 95% CI, 95% confidence interval.** *P*<0.01.

Data fitting detected a linear correlation between the indexes of soil aggregates and the fractal dimension (Fig. 6).

## Discussion

Results of this study demonstrated that application of crop straws had positive effects on the soil physico-chemical properties. On the other hand, there are Loess plateau regions at northwest China with intensive cultivation systems and poor soil management strategies. Therefore, crop residual management is very important for preserving natural ecosystems [38]. Another problem for this area is conventional tillage that reduces the soil water storage and destroys the aggregates and soil structure [39] which prompts deterioration of crop yield.

The results indicated that the SOC storage increased significantly in the 0–60 cm layers after three years of straw incorporation (Fig. 2). Rasmussen and Collins [40] reported that the soil organic matter content was strongly related to the amount of residues added and only weakly related to the type of residue applied. The H and M treatments were significantly different from the L throughout the three years and the soil organic matter level increased as the straw incorporated and decomposed [20], which effectively mitigated the loss of soil organic carbon from in the agroecosystem caused by intensive cropping [41]. The SOC storage decreased gradually in all the treatments with the soil layer depth because the degree of straw incorporation was lower in the deeper layers compared with the surface layers (0–20 cm) of the soil [42]. This was because the amount of straw incorporated in the topsoil was greater than that in the deeper layers [40], [43].

Soil aggregates are the basic units of the soil structure and they are composed of primary particles and binding agents [12]. They are also necessary soil conditions for high crop yields [44]. Conventional tillage disturbs the soil and increase the effects of drying–rewetting and freezing–thawing, which increases the susceptibility of the macroaggregate (>0.25 mm) to disruption [45]–[47]. Pinheiro *et al.* [48] showed that soil exposure with tillage and the lack of residue inputs led to a decline in aggregation and organic carbon, both of which made the soil susceptible to erosion. Our study showed that the straw incorporation of straws determined significantly more and larger soil aggregates than CK, thus indicating an improvement of soil physical quality. This may have been attributable to the significant increase in the SOC storage (an average increase by 8.21–21.40% in 2010), the lower soil bulk density (an average decreased by 1.80–4.13% in 2010, data no shown) [49], and increased soil porosity (an average increased by 1.70–3.90% in 2010, data no shown) [50] after straw incorporation. It also stimulated the activity of soil microorganisms [51] and an abundance of polyose metabolites were produced during the straw decay process [52]. These soil physical and chemical conditions may have accelerated the incorporated SOM decomposing process and increased soil aggregation [24].

Soil aggregate structural stability is widely recognized as a key indicator of soil quality, which is closely related to a number of soil properties, processes, and functions, e.g., the quantity and composition of SOM [48], infiltration capacity [53], soil biotic activity [54] and the resistance to erosion [55], [56]. Wei *et al.* [57] showed that the addition of crop residues was the most effective measure for increasing the rhizosphere aggregate stability. Sonnleitner *et al.* [24] and Karami *et al.* [41] also found that straw application improved the aggregate stability and other soil properties. In our study, the soil aggregate stability of the straw incorporation treatments were significantly higher than CK in 2010, and it was decreased with the soil layer depth. These results agreed with studies by Tripathy and Singh [42] and Karami *et al.* [41]. Our results also indicated that straw incorporation was positively related to the physical protection of organic matter [20] and an increased aggregate quantity [58], but it also improved the soil aggregate stability [20] and reduced soil degeneration [22], [23].

Many studies have shown that soil is a porous medium with fractal characteristics [59]–[60]. Thus, fractal theory can be used to describe the complex characteristic of soil structure [61]. Castrignanò and Stelluti [62] reported that a higher fractal dimension indicated the heavier texture of a soil and its inferior permeation properties. This showed that fractal theory is an effective method to describing the soil aggregate distribution [37] and changed with different levels of straw incorporation [31]. The fractal dimensions of the 0–40 cm layers with the four treatments after three years were ranked in the order: H<M<L<CK and the three straw incorporation treatments were significantly different from CK. These results agreed with Zhang *et al.* [37] and Zhang *et al.* [31]. The low values of *D* indicated a size distribution dominated by a large number of macroaggregates (>0.25 mm) [59], [63]. This improvement in the fractal dimension may have been accelerated by the incorporation of straw, which improved the soil structure, increased the SOM content and microbial activity [64], and significantly increased the mount and size of soil aggregates [37]. Our results indicated there were significant improvements in the soil macroaggregates and the aggregate structure after straw incorporation [20].

## Conclusion

The incorporation of different amount of straw significant increased the *SOC* storage, >0.25 mm macroaggregates, *MWD* and *GMD* in a semiarid soil. The *SOC* storage, >0.25 mm macroaggregates, *MWD* and *GMD* also increased with higher straw incorporation rates. The fractal dimension decreased with increasing straw incorporation rates. Therefore, the incorporation of straw into the soil in semiarid areas is an effective practice for improving the soil aggregate content and stability.
